# The Impact of Documented Electronic Referrals to the Smoking Cessation Clinic Before Discharge From Medical Wards on Patients’ Adherence to Clinic Follow-Up

**DOI:** 10.7759/cureus.108002

**Published:** 2026-04-29

**Authors:** Naveed Uddin, Muhammad Sohaib Ejaz Khan, Alia Abotaleb, Saleh Y Alhedyan, Usman Ghani, Dheyya F Alnajar, Yara Alqurashi

**Affiliations:** 1 Internal Medicine, King Fahad Armed Forces Hospital, Jeddah, SAU; 2 Medicine, King Fahad Armed Forces Hospital, Jeddah, SAU

**Keywords:** cigarette smoking, clinic adherence, electronic referral systems, follow-up appointment, post-discharge

## Abstract

Objective

The objective of this study is to evaluate whether the implementation of a mandatory electronic inpatient referral pathway to the Smoking Cessation Clinic (SCC) improves referral completion and post-discharge clinic attendance.

Methods

A six-month prospective quality improvement study was conducted from May to October 2025 in the Department of Medicine at King Fahad Armed Forces Hospital (KFAFH), Jeddah. This study aimed to identify hospitalized smokers, assess readiness to quit, and implement documented electronic referrals to the Smoking Cessation Clinic (SCC) prior to discharge. Baseline data were collected from 27 medical inpatients with a smoking history who agreed to referral. The intervention incorporated systematic smoking status documentation, motivational counseling using the “5 As” framework, provider education, and audit-feedback cycles. Clinic attendance was assessed within 90 days of discharge.

Results

Among the 27 admitted patients with a six-month follow-up, 100% were identified as current smokers. Readiness and motivation to quit were assessed in all the patients. One hundred percent (27/27) were referred to a Smoking Cessation Clinic, with 100.0% (27/27) of referrals made electronically. A documented appointment was booked for all 27 patients prior to discharge. The primary outcome, clinic attendance within 90 days, was achieved in 100.0% (27/27) of patients. The end-to-end success rate (identification → assessment → referral → appointment → attendance) was 100.0%.

Conclusions

Embedding a mandatory electronic smoking cessation referral pathway within inpatient workflows resulted in complete adherence to referral and follow-up processes. Integration within the electronic health record (EHR), pre-booked appointments, and provider engagement were key enablers. This model is scalable and supports the implementation of a hospital-wide mandatory smoking cessation pathway for admitted smokers.

## Introduction

The tobacco epidemic is one of the biggest public health threats the world has ever faced, responsible for over seven million deaths annually, as well as disability and long-term suffering from tobacco-related diseases [1]. However, cigarette smoking is the most common form of tobacco use worldwide and remains a leading preventable cause of cardiovascular disease, chronic respiratory disease, malignancy, and premature mortality worldwide [2]. Smoking doubles the risk of premature cardiovascular mortality [3], substantially increasing readmissions and healthcare utilization [4].

Smoking cessation interventions during hospitalization have been shown to improve quit rates, particularly when linked to post-discharge support [5]. Barriers to referral include a lack of electronic mechanisms and nonmandatory counseling in care plans [6]. Research suggests that patients who have appointments scheduled for them are more likely to attend Smoking Cessation Clinics (SCC) compared to those who are advised to book their own appointments. A study published in The Journal of the American Medical Association (JAMA) found that patients who received facilitated referrals (i.e., had appointments scheduled for them) had a significantly higher attendance rate at Smoking Cessation Clinics compared to those who received traditional referrals (i.e., were advised to book their own appointments) [7]. A systematic review and meta-analysis published in the Cochrane Database of Systematic Reviews found that interventions that included facilitated referral to smoking cessation services increased the likelihood of patients attending counseling sessions and quitting smoking [8]. In terms of statistical difference, a study published in the Journal of General Internal Medicine found that patients who received facilitated referrals had a 23.4% attendance rate at Smoking Cessation Clinics, compared to 6.1% for patients who received traditional referrals (p < 0.001) [9].

Despite strong guideline recommendations advocating the systematic identification and treatment of tobacco dependence in hospitalized patients, implementation remains inconsistent [9,10]. At our institution, inpatient referral to the SCC is neither electronic nor mandatory, even among high-risk patients. Patients are verbally advised at discharge to self-arrange SCC appointments through primary care services, a process associated with poor attendance and loss to follow-up [11,12].

This study aimed to identify hospitalized smokers, assess readiness to quit, and implement a documented electronic referral pathway to the SCC prior to discharge to improve clinic attendance and engagement with evidence-based cessation services.

Preliminary data analysis revealed the following: A baseline survey of 27 medical inpatients with smoking history showed that 78.6% were current smokers (84.5%, men; 15.5%, women), 52.4% daily smokers, 31.7% occasional smokers, and 15.9% former smokers; 70.1% had failed prior quit attempts; and 14.6% never tried. Despite 65.1% receiving quit advice, only 12.6% were ever referred to SCC. The mean pack-years were 28.4 ± 18.2; 30.8% started before the age of 18. Common comorbidities were hypertension (60.8%), diabetes (48.6%), and dyslipidemia (41.2%).

The baseline findings demonstrated low referral rates (12.6%) despite frequent counseling, highlighting a gap in linkage to smoking cessation services and providing a comparator for evaluating the impact of the current electronic referral intervention.

Referral completion and clinic attendance are recognized indicators of high-quality transitional care [13,14]. Facilitated referrals with scheduled appointments significantly improve clinic attendance compared to patient-initiated booking, particularly when supported by electronic health record (EHR)-based workflows [15]. Improving these metrics has the potential to reduce smoking-related readmissions, improve patient safety during the post-discharge period, and optimize healthcare resource utilization.

## Materials and methods

Study design

This prospective quality improvement study was conducted in the medical wards of King Fahad Armed Forces Hospital (KFAFH), Jeddah, Saudi Arabia, using the Plan-Do-Study-Act (PDSA) methodology in accordance with the Standards for QUality Improvement Reporting Excellence (SQUIRE) 2.0 principles [13]. The intervention period spanned six months, with follow-up extending to 90 days after hospital discharge.

Study population

All adult patients admitted to the medical wards during the study period were screened for a history of tobacco use. Patients who did not meet the inclusion criteria, declined referral, or had exclusion criteria (e.g., critical illness or cognitive impairment) were excluded. Of the patients approached (n = 31), 27 consented to participate and were enrolled in the study, while four declined enrollment. The inclusion and exclusion criteria used for patient selection are summarized in Table 1.

**Table 1:** Inclusion and Exclusion Criteria for Patients Included in the Study

Category	Criteria
Inclusion criteria	Age ≥ 18 years
	Current or previous smoking history documented in the electronic health record
	Willingness to receive a referral to the Smoking Cessation Clinic
Exclusion criteria	Critical illness requiring intensive care admission
	Cognitive impairment precluding informed participation
	Patient refusal to participate or be referred

A total of 27 hospitalized smokers were enrolled in the intervention. The intervention consisted of several integrated components. Smoking status was documented as a vital sign within the electronic health record. Patients received brief motivational counseling based on the “5 As” framework (ask, advise, assess, assist, and arrange). Electronic referrals to the Smoking Cessation Clinic (SCC) were generated before hospital discharge, with appointments scheduled in advance. Educational materials were provided to patients who declined referral. In addition, provider education sessions and weekly audit-feedback cycles were conducted to reinforce adherence to the smoking cessation pathway.

The primary outcome of the study was patient attendance at the SCC within 90 days of hospital discharge. Secondary outcomes included the completion of smoking status documentation in the electronic health record, the assessment and documentation of patients’ readiness and motivation to quit smoking, successful electronic referral to the SCC, and the scheduling of SCC appointments prior to hospital discharge.

Data collection

Data were extracted from the electronic health record and SCC appointment logs.

Statistical analysis

Data were analyzed using Microsoft Excel (Microsoft Corporation, Redmond, WA) for descriptive statistics. Results are reported as frequencies and percentages. Given the quality improvement design and small sample size, no formal hypothesis testing was performed.

Ethical approval

Ethical approval was obtained from the Research Ethics Committee of King Fahad Armed Forces Hospital (KFAFH), Jeddah (approval number: REC 897, dated January 15, 2026). The study was conducted in accordance with institutional ethical guidelines and the principles of the Declaration of Helsinki.

## Results

**Figure 1:**
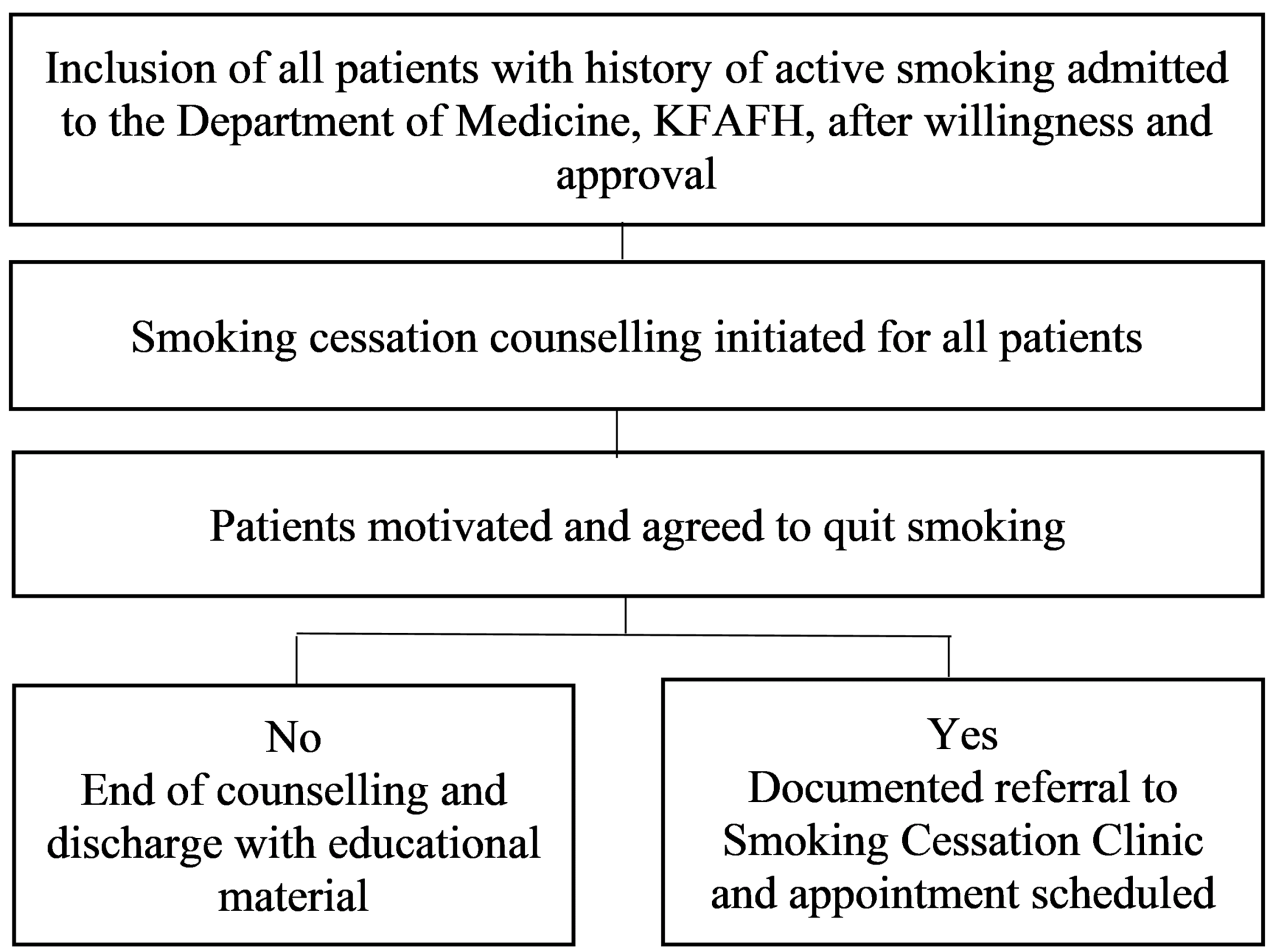
Flow Diagram Illustrating the Identification of Hospitalized Smokers, the Assessment of Readiness to Quit, Electronic Referral to Smoking Cessation Clinic, and Follow-Up Attendance Within 90 Days After Hospital Discharge KFAFH: King Fahad Armed Forces Hospital

Admitted patients were screened during the study period. During the study period, 31 patients were approached, of whom 27 consented to participate, and four declined enrollment. After applying inclusion and exclusion criteria and accounting for patient refusal, 27 patients were enrolled in the intervention. All enrolled patients completed every step of the smoking cessation pathway (Table 2). The patient selection process is illustrated in Figure 1. Electronic referral was associated with 100% clinic attendance, consistent with evidence demonstrating improved adherence through facilitated referral models [8,11].

**Table 2:** Completion of Smoking Cessation Referral Pathway SCC: Smoking Cessation Clinic

Pathway Step	n/N	Percentage (%)
Smokers identified at admission	27/27	100
Readiness and motivation assessed	27/27	100
Referred to the SCC	27/27	100
Referral completed electronically	27/27	100
SCC appointment scheduled prior to discharge	27/27	100
Attended SCC within 90 days	27/27	100
Full pathway completion	27/27	100

A total of 27 hospitalized patients with a documented history of tobacco use who met the eligibility criteria were included in the study. All enrolled participants were identified as current smokers at the time of hospital admission. Smoking status was systematically documented in the electronic health record for all patients, and readiness and motivation to quit smoking were assessed during the hospital stay.

The implementation of the electronic referral pathway resulted in complete adherence to each step of the smoking cessation process. All 27 patients (100%) were successfully referred to the Smoking Cessation Clinic (SCC), and every referral was completed electronically through the hospital’s electronic health record system. Furthermore, appointments at the SCC were scheduled for all patients prior to hospital discharge. As shown in Table 2, all enrolled patients attended their scheduled smoking cessation clinic appointment within 90 days following discharge, resulting in a 100% completion rate across the entire care pathway.

Baseline smoking characteristics of the study participants are summarized in Table 3. Cigarette smoking was the primary tobacco product used by all participants. The majority of patients reported initiating smoking between 15 and 20 years of age (52%, n = 14), followed by 21-30 years (33%, n = 9) and 31-40 years (15%, n = 4). Regarding smoking intensity, 44% (n = 12) of the participants reported smoking between one and one-and-a-half packs of cigarettes per day, while 30% (n = 8) smoked between half and one pack daily. A smaller proportion (19%, n = 5) reported smoking two to three packs per day. The mean cumulative smoking exposure was 25.6 ± 14.2 pack-years. In addition, most participants had previously attempted to quit smoking, with 70% (n = 19) reporting multiple quit attempts, 22% (n = 6) reporting one or two attempts, and only 7% (n = 2) reporting no prior quit attempts.

**Table 3:** Baseline Smoking Characteristics of Enrolled Patients (N = 27)

Variable	Category	n	%
Primary tobacco product	Cigarettes	27	100
Age at smoking initiation	15-20 years	14	52
	21-30 years	9	33
	31-40 years	4	15
Cigarettes per day	½-1 pack	8	30
	1-1 ½ packs	12	44
	2-3 packs	5	19
Pack-years	Mean ± SD	25.6 ± 14.2	-
Previous quit attempts	Multiple attempts	19	70
	1-2 attempts	6	22
	Never attempted	2	7

Attendance at the Smoking Cessation Clinic according to the referral method is presented in Table 4. All patients who received electronic referrals attended their scheduled clinic appointments, resulting in an attendance rate of 100%. This finding highlights the effectiveness of the facilitated electronic referral system in improving patient engagement with smoking cessation services.

**Table 4:** SCC Attendance by Referral Method SCC: Smoking Cessation Clinic

Referral Method	Attendance at the SCC (n/N)	%
Electronic referral	27/27	100
Nonelectronic referral/verbal advices	0/27	0

Patient-reported motivations for smoking cessation are presented in Table 5. The most commonly reported reason for attempting to quit smoking was concern about personal health, reported by (74%, n = 20). Other commonly cited motivations included the desire to become smoke-free (44%, n = 12), family considerations (37%, n = 10), odor or personal discomfort related to smoking (30%, n = 8), and broader social reasons (26%, n = 7).

**Table 5:** Patient-Reported Reasons for Smoking Cessation (N = 27)

Reasons	n	%
Health concerns	20	74
Desire to be smoke-free	12	44
Family considerations	10	37
Odor/personal discomfort	8	30
Social reasons	7	26

Overall, the intervention achieved complete adherence across the smoking cessation referral pathway, including the identification of smokers, the assessment of readiness to quit, electronic referral to the clinic, appointment scheduling prior to discharge, and post-discharge clinic attendance.

## Discussion

Among the hospitalized smokers who accepted referral to the Smoking Cessation Clinic (SCC), 27 patients were enrolled in the project. The intervention achieved universal smoker identification (100%), complete readiness and motivation assessment (100%), and high referral rates (100%, all electronic), representing secondary process outcomes. The primary outcome, clinic attendance within 90 days of discharge, was achieved in all enrolled patients (100%). This 100% end-to-end success demonstrates the feasibility of embedding evidence-based tobacco dependence treatment into routine admission workflows.

The observed 100% clinical attendance rate may be explained by the facilitated nature of the intervention, including mandatory electronic referrals, pre-scheduled appointments prior to discharge, and active provider engagement. These system-level changes likely minimized common barriers such as missed bookings, the lack of follow-up coordination, and patient drop-off after discharge. However, these results should be interpreted within the context of a small, selected cohort of patients who accepted referral, which may overestimate real-world adherence rates.

The model is highly cost-effective through expected financial savings from fewer readmissions and is scalable to other high-risk behaviors, although sustainability requires institutional policy mandating referrals for smokers with not only the cardiovascular or pulmonary disease but also any other medical background. Based on the results of our improvement project, implementing a mandatory "smoking cessation pathway" in the admission bundle for all patients across the hospital could significantly boost attendance at Smoking Cessation Clinics. Based on the existing literature, hospital-based smoking cessation interventions have been associated with a reduction in smoking-related hospital admissions due to conditions such as chronic obstructive pulmonary disease (COPD), thromboembolic diseases, and heart failure. Furthermore, long-term smoking cessation is known to have an impact on decreasing the incidence of smoking-related malignancies. However, these clinical outcomes were not directly measured in the present study. The financial implications would also be notable, with potential cost savings for both patients and the healthcare system, as suggested by prior studies.

A study on the Ottawa Model for Smoking Cessation found that hospital-initiated smoking cessation interventions reduced all-cause readmissions, 30-day readmission rates decreased by 6.1% (13.3% versus 7.1%), 1-year readmission rates decreased by 11.7% (38.4% versus 26.7%), and 2-year readmission rates decreased by 11.6% (45.2% versus 33.6%). Significant reductions were observed at all time points in smoking-related readmissions. Additionally, emergency department visits decreased by 4.5% (20.9% versus 16.4%) within 30 days.

The financial benefits of hospital-based smoking cessation interventions are also well established. According to the American Lung Association estimates, for every dollar spent on tobacco cessation treatments, there is a potential return on investment of $1.26. Annual savings cause a reduction in relation to direct healthcare expenditures of approximately $275 million, in addition to workplace productivity losses of nearly $436 million, for a combined total potential annual savings of $711 million.

Regarding smoking-related malignancies, quitting smoking can significantly reduce the incidence and associated financial burden. Smoking cessation interventions can reduce the risk of developing smoking-related cancers, which are a major driver of healthcare costs, with savings estimated at $301 billion annually to the US economy due to smoking-related healthcare expenditures and productivity losses.

Mandating a hospital-wide "Smoking Cessation Pathway" will definitely extrapolate into financial benefits. A second, more extensive, and prolonged phase of this project can be initiated in the next fiscal year to unleash the promised benefits. The sustained impact of the intervention was evident across all components of the pathway. There was consistent 100% identification of smoking status at admission, ensuring no missed opportunities for intervention. Assessment of readiness and motivation to quit was also maintained at 100%, effectively embedding motivational counseling into routine inpatient care. Referral rate to Smoking Cessation Clinic remains universally high, with 100% of smokers being referred and 90% of these referrals completed electronically, demonstrating durable system-level integration. Appointment booking was equally successful, providing effective linkage between inpatient care and outpatient services. Importantly,100% sustained clinic attendance reflects robust long-term patient engagement beyond hospital discharge.

Limitations

This study was conducted at a single center with a small sample size, which may limit the generalizability of the findings to other institutions with different populations. Selection bias is present, as only patients who agreed to referral were included. The study design did not include a control group, and follow-up was limited to 90 days, reflecting a short-term evaluation. In addition, smoking cessation outcomes were not directly measured, and self-reported abstinence may not fully reflect long-term clinical impact. This limitation underscores the need for broader implementation and longer-term evaluation.

## Conclusions

Implementation of a mandatory inpatient smoking cessation pathway with electronic referral and pre-booked appointments significantly improved clinic attendance. Scaling this intervention hospital-wide may reduce smoking-related readmissions, long-term morbidity, and healthcare costs. Further longitudinal evaluation is warranted to assess sustained abstinence and economic impact. The E-referral system has significant potential for the future of E-health, but more work needs to be done to understand how to maximize referral and subsequent participation.
